# Leaving the Past Behind

**DOI:** 10.1371/journal.pgen.1000248

**Published:** 2008-10-31

**Authors:** E. Jean Finnegan, Emma Whitelaw

**Affiliations:** 1 Commonwealth Scientific and Industrial Research Organisation (CSIRO), Plant Industry and Climate Adaptation Flagship, Canberra, Australia; 2 Queensland Institute of Medical Research, Brisbane, Australia

There is considerable interest from the wider scientific community in the heritability of epigenetic states across generations, and this has arisen as a result of a series of studies in mice [1],[2], flies [3], plants [4],[5], and yeast [6] over the past decade. These studies have identified genetic elements at which epigenetic states appear to be inherited through meiosis. The Lamarckian implications of these findings are hard to avoid. Transgenes, transposons, and other “foreign DNA” appear to be particularly prone to transgenerational epigenetic inheritance (reviewed in [7]). In this issue of *PLoS Genetics*, Singh et al. [8] describe the identification of a locus in the genome of maize at which a transposon, silenced by an RNAi-based mechanism, becomes reactivated over subsequent generations. This article reports an activating “position effect,” i.e., an integration site that is associated with the reversal of a previously established silent state in plants.

**Figure 1:**
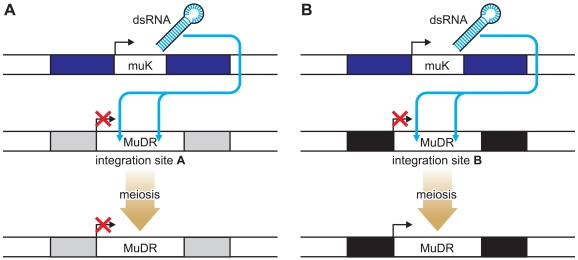
Locus-Specific Reactivation of the MuDR Transposon. When *MuDR* and *Muk* are both present in one plant, the *MuDR* elements become epigenetically silenced as a result of a long hairpin RNA molecule produced from *Muk* that acts in *trans* to initiate DNA methylation of *MuDR* elements. At most loci, once the *MuDR* has been silenced it remains so even after *Muk* segregates away (A). In contrast (B), when inserted within the *Hemera* (black bar) locus, *MuDR* was reactivated in progeny plants that did not inherit *MuK*.

The authors have devised a clever system for studying position effects that involves a single transposon, *MuDR*, and a variant of *MuDR*, called *Mu* killer (*Muk*) [8]. The integration site of the *MuDR* can be altered by transposition. When *MuDR* and *Muk* are both present in one plant, the *MuDR* elements become epigenetically silenced as a result of a long hairpin RNA molecule produced from *Muk* that acts in *trans* to initiate DNA methylation of *MuDR* elements (Figure 1). Once the *MuDR* has been silenced, it generally remains so even after *Muk* segregates away in subsequent generations (Figure 1A). This is consistent with observations made by others studying the activity of endogenous genes or transgenes that have been silenced by RNA-directed mechanisms in plants [5],[9],[10] and with transgenes in mice [11]. However, at one particular integration site, they found that the opposite was true. Following the loss of *Muk*, the *MuDR* element reactivated, an event associated with loss of DNA methylation (Figure 1B). The integration site in this case turns out to be the 5′ untranslated region (UTR) of a gene of unknown function, designated *Hemera* [8].

Plant transposons frequently insert near or within transcribed genes, so what is special about this case? It is not known whether insertion of the transposon blocks *Hemera* activity, but if it does, then a trivial explanation for the reactivation of *MuDR* is that *Hemera* plays a role in maintaining silencing of targets of the RNA-directed DNA methylation pathway. A more likely and more interesting scenario is that reprogramming of *Hemera* during gamete formation or during the early stages of development of the subsequent embryo is associated with reactivation of the *MuDR* inserted within the 5′ UTR of this gene. The authors note that *MuDR* has inserted adjacent to a GA-rich sequence and suggest that this may be important for the reprogramming of both *MuDR* and *Hemera* during their passage to the next generation. This hypothesis could be readily tested using transgenic approaches to alter the sequences that flank *MuDR* in *Hemera*.

**Figure 2:**
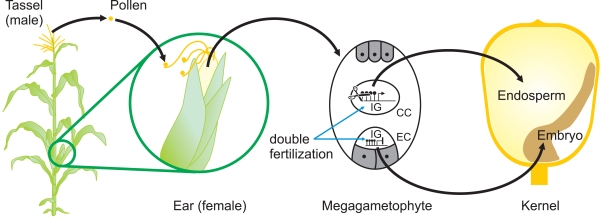
Sites of Potential Epigenetic Reprogramming during Maize Reproduction. The reproductive organs, the ear, and the tassel of a maize plant arise when vegetative meristems differentiate to become inflorescence meristems. Pollen, formed in the tassel, falls onto the silks where it germinates. A pollen tube, containing two identical haploid sperm nuclei, grows down the silk until it reaches the megagametophyte containing the haploid egg cell (EC) and the diploid central cell (CC). One sperm nucleus fuses with the EC and the other fuses with the CC (double fertilization), giving rise to the zygote (diploid) and endosperm (triploid), which provides nutrients to the developing embryo. Epigenetic reprogramming that removes methylcytosine from the control regions of imprinted genes occurs in the CC but not in the EC, leading to differential expression of these genes in endosperm [16]. It is likely that other, as-yet uncharacterised, epigenetic reprogramming events occur during pollen or egg cell formation as well as during early stages of embryo or endosperm development.

To plant epigeneticists, who focus mainly on transposons and transgenes, the reactivation of a silenced *MuDR* is a surprise. But to mammalian epigeneticists, it is not. In mice, for example, it is widely accepted that *cis*-acting sequences, e.g., promoters, are reprogrammed each generation so that the cells of the preimplantation embryo can acquire pluripotency. Indeed, for the mammalian epigeneticist, transgenerational epigenetic inheritance is the exception rather than the rule. Even the described cases of transgenerational epigenetic inheritance in mammals actually display considerable reprogramming of epigenetic state from generation to generation. The *agouti viable yellow* allele and the *axin-fused* allele are two well-characterised examples [1],[12]. It seems likely that there is epigenetic reprogramming of endogenous plant genes to ensure that the normal program of plant development is reiterated each generation (Figure 2), no matter what conditions the parental plant experienced. Indeed, it has recently been shown that the vernalization-induced epigenetic repression of the *Arabidopsis FLC* gene is reversed during pollen development or, when inherited through the maternal gamete, in the globular embryo [13].

So what does this new finding tell us? It reaffirms the idea that the molecular mechanisms involved in the permanent silencing of foreign DNA have evolved from the mechanisms required for the successful development of an embryo. Consistent with this idea, random mutagenesis screens for modifiers of position effect variegation carried out in both *Drosophila* [14] and mouse [15] have found that most genes identified play critical roles in development. It has been difficult for plant biologists to study the developing embryo, because it is surrounded by developing endosperm and is embedded in the somatic tissue of the parent plant. In contrast preimplantation mouse embryos develop as unattached entities that can be flushed out of the uterus. As plant biologists acquire better methods of studying the zygote as it develops, they are likely to find more genetic elements of this type. For development to work at all, the genomes of multicellular organisms must leave the past behind.
